# Increased temporal stride variability contributes to impaired gait coordination after stroke

**DOI:** 10.1038/s41598-022-17017-1

**Published:** 2022-07-25

**Authors:** Prakruti Patel, Diana Enzastiga, Agostina Casamento-Moran, Evangelos A. Christou, Neha Lodha

**Affiliations:** 1 Movement Neuroscience and Rehabilitation Laboratory, Department of Health and Exercise Science, Colorado State University, Fort Collins, CO 80523 USA; 2 Department of Applied Physiology and Kinesiology, University of Florida, Gainesville, FL USA

**Keywords:** Neurology, Neurological disorders, Stroke

## Abstract

Heightened motor variability is a prominent impairment after stroke. During walking, stroke survivors show increased spatial and temporal variability; however, the functional implications of increased gait variability are not well understood. Here, we determine the effect of gait variability on the coordination between lower limbs during overground walking in stroke survivors. Ambulatory stroke survivors and controls walked at a preferred pace. We measured stride length and stride time variability, and accuracy and consistency of anti-phase gait coordination with phase coordination index (PCI). Stroke survivors showed increased stride length variability, stride time variability, and PCI compared with controls. Stride time variability but not stride length variability predicted 43% of the variance in PCI in the stroke group. Stride time variability emerged as a significant predictor of error and consistency of phase. Despite impaired spatial and temporal gait variability following stroke, increased temporal variability contributes to disrupted accuracy and consistency of gait coordination. We provide novel evidence that decline in gait coordination after stroke is associated with exacerbated stride time variability, but not stride length variability. Temporal gait variability may be a robust indicator of the decline in locomotor function and an ideal target for motor interventions that promote stable walking after stroke.

## Introduction

Variability in motor output is a fundamental characteristic of human motor performance. Motor variability is evident in discrete tasks such as kicking a ball as well as in continuous tasks such as walking^1,2^. Human gait represents a highly skilled form of movement that is characterized by relatively low fluctuations in timing and positioning of consecutive steps in healthy adults. It is theorized that a lower level of gait variability enables better adaptability of the walking pattern to the environmental changes^3,4^. However, heightened gait variability during minimal physical or task constraints is linked with poor behavioral outcomes such as accidental falls, decline in cognition, and mental health^4–10^. As such, a growing body of literature identifies gait variability as a clinical biomarker of ageing or pathology. Individuals with gait dysfunction due to stroke have a greater amount of spatial and temporal gait variability relative to controls^11,12^. However, unlike the ageing literature, the implication of increased gait variability after stroke is not well understood. Our study focuses on the consequence of increased gait variability in stroke on the coordination between lower limbs, an important aspect of safe mobility.

Safe mobility relies on continuous and rhythmical coordination between lower extremities such that both legs alternate in swing and stance phase during walking^13–15^. Poor gait coordination may limit the ability to adapt walking to different environments or to sudden perturbations^16^. Consequently, older adults with poor gait coordination report higher incidence of limitations in walking and transfers^17–19^. Even though age-related changes in gait coordination have been well studied, few studies have directly investigated deficits in lower limb coordination during walking after stroke. Some evidence suggests that individuals with stroke exhibit lower limb incoordination during voluntary ankle movement tasks and treadmill walking^20,21^. To date, the influence of stroke on lower limb coordination during overground walking remains unclear.

Given that human gait is a highly repeatable form of movement, a higher amount of variability in gait parameters may reflect an error in selection or execution of motor programs, interfering with skilled walking performance^22^. As gait coordination involves simultaneous, synchronous movement of each lower extremity, increased fluctuations in the timing and positioning of individual foot may affect coordination between the two lower limbs. However, empirical evidence in healthy young and older adults suggests that temporal gait variability is not related to gait coordination, pointing to the possibility of different control mechanisms for these variables in the absence of gait disorders^13,14^. In contrast, in individuals with gait dysfunctions such as Parkinson’s Disease, multiple sclerosis, and fibromyalgia, poor gait coordination between lower limbs and increased stride-to-stride variability seem to coexist^13,23,24^. A previous study in individuals with Parkinson’s disease (PD) reported that increased stride time variability was related to gait incoordination in PD^13^. Likewise, in people with multiple sclerosis, gait incoordination was related to increased temporal gait variability regardless of the severity of disease^23^. These studies point to the possibility that higher levels of fluctuations in timing and positioning of individual limbs may potentially impact coordination between the two limbs during walking. However, the empirical evidence supporting this hypothesis in stroke survivors is lacking.

Here, we investigate the effect of spatial and temporal gait variability on coordination between lower limbs during overground walking in stroke survivors. To assess lower limb coordination during walking, we measured phase coordination index (PCI) that quantifies the accuracy and consistency of anti-phase leg movements during walking^13^. Individuals with stroke often modulate their gait by altering spatial or temporal strategies to compensate for sensorimotor impairments of the affected limb. Evidence suggests that the deficits in spatial and temporal gait parameters may exist independent of each other in stroke. For example, stroke survivors with mild-moderate impairments showed swing time asymmetry but no step length asymmetry^25^. Another study showed that after body-weight support treadmill training, stroke survivors modulated stride length (spatial gait outcome) but not cadence (temporal gait outcome) to improve gait speed^26^. These studies suggest that stroke survivors may differentially modulate spatial and temporal gait parameters to alter the overall walking outcome. Likewise, gait coordination after stroke may be affected by inconsistency in stride time or stride length.

The purpose of our study is determine the relationship between gait variability and gait coordination, and the relative contribution of stride time (temporal) variability versus stride length (spatial) variability to gait coordination in stroke survivors during overground walking. We hypothesized that increase in temporal and spatial gait variability will relate to poor gait coordination in stroke; however, the relative contribution of spatial versus temporal gait variability to gait coordination will differ. Prior work suggests that individuals with stroke show an increased risk for falls during activities that require coordination between the lower extremities such as walking, turning, and position transfer^27–29^. Recent work from others and our group suggests that gait variability can be improved in people with gait dysfunction^30,31^. Thus, identifying potentially modifiable biomechanical factors that contribute to deficits in gait coordination can facilitate the development of effective gait interventions that promote safe mobility after stroke.

## Results

### Gait variability

**Figure 1:**
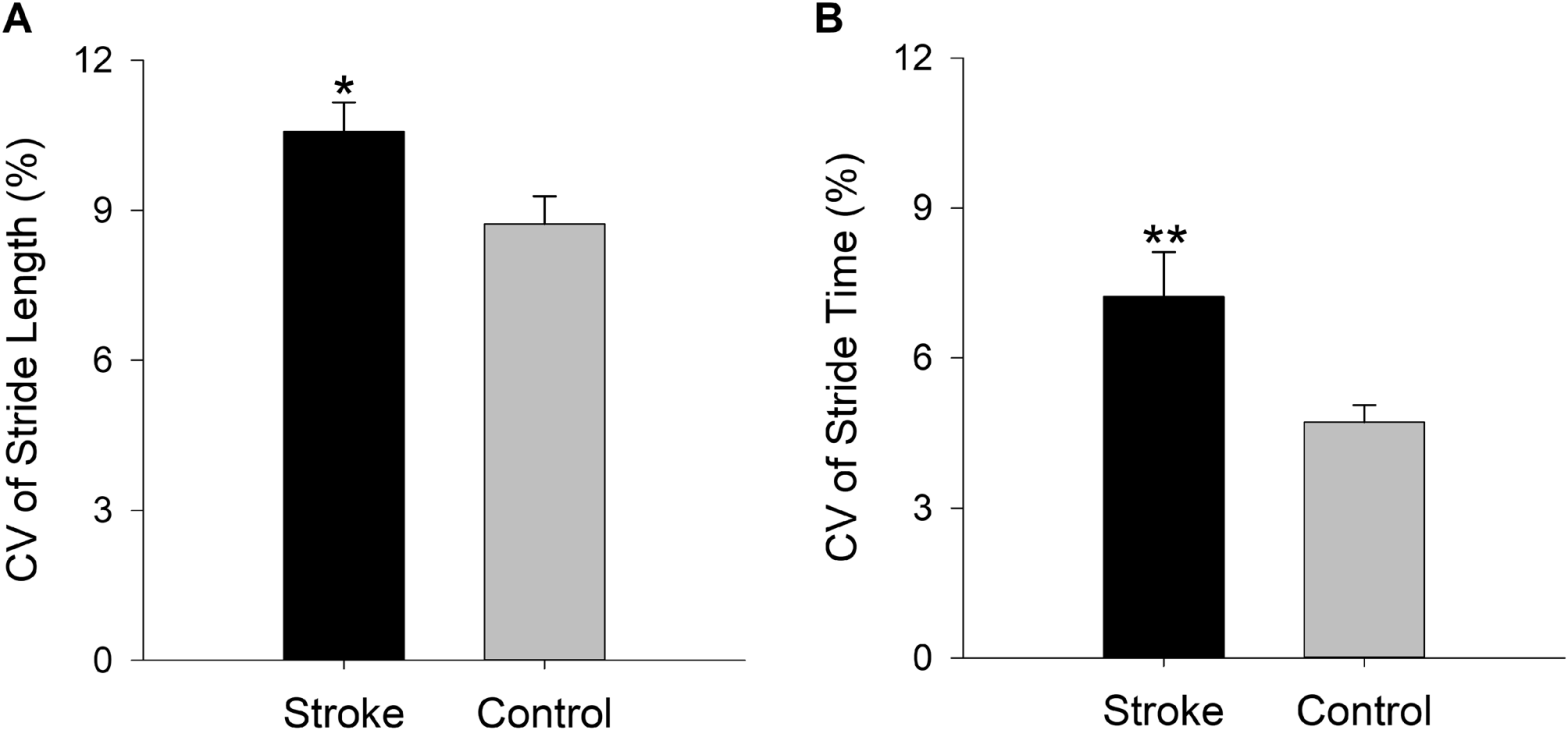
The impact of stroke on spatial and temporal gait variability: The gait variability measures were averaged across the two legs. The stroke group showed increased stride length variability (**A**) and increased stride time variability (**B**) compared with the control group. *p < 0.05. **p < 0.01.

The stroke group showed significantly greater stride length variability relative to the control group (*t*_|40|_= 2.31, *p* = 0.02; Cohen’s *d* = 0.71, Fig. 1A). The stroke group also showed greater stride time variability compared with the control group (*t*_|40|_= 2.80, *p* < 0.01; Cohen’s* d* = 0.86 Fig. 1B). The stride length variability was increased by 21.33% and the stride time variability was increased by 53.17% in the stroke group. The stroke group showed reduced gait speed compared with the controls (*t*_|40|_= −3.04, *p* = 0.004, Cohen’s *d* = 0.95; Table 1). Gait speed was reduced by 18.26% in the stroke group.

**Table 1:** Participant characteristics.

Characteristics	Stroke (n = 21)	Control (n = 21)	*t* statistic	*p* value
Age (years)	62.12 ± 13.40	63.90 ± 10.99	−0.46	0.64
Sex (male/female), *N*	11/10	10/11	n/a	n/a
Hemiparetic side (left/right)	9/12	n/a	n/a	n/a
FAI (/45)	29.52 ± 7.41	32.24 ± 4.43	−1.44	0.15
Gait speed (m/s)	0.94 ± 0.29	1.15 ± 0.11	−3.04	0.004
FMA-LE (/34)	27.38 ± 6.08	n/a	n/a	n/a

All data is presented as mean ± standard deviation.

*FAI* Frenchay activities index, *FMA-LE* Fugl-Meyer assessment of lower extremity, *n/a* not applicable.

### Lower limb coordination during walking

**Figure 2:**
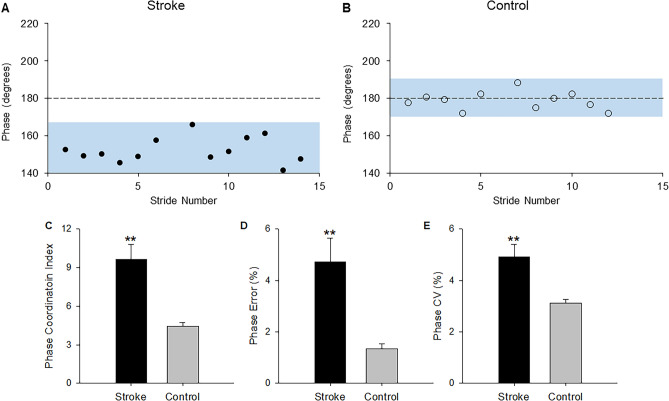
The impact of stroke on bilateral gait coordination: the phase values from a representative walking trial of (**A**) a stroke participant and (**B**) a control participant. The dashed line represents the ideal phase of 180° during walking. The shaded region represents the variability in phase values across the strides. The individual with stroke showed greater deviation of the phase from the 180° and higher variability of phase across consecutive strides relative to the control. Compared with the control group, the stroke group showed significantly increased (**C**) phase coordination index, (**D**) phase error relative to the ideal phase of 180°, and (**E**) coefficient of variation (CV) of phase during overground walking. **p < 0.01.

Figure 2A,B shows the phase values for consecutive strides from a representative walking trial of a stroke and a control participant. The stroke group had significantly greater PCI compared with the control group (*t*_|40|_= 3.95, *p* < 0.001; Cohen’s* d* = 1.22; Fig. 2C). The PCI was higher by 127.65% in the stroke group. Likewise, the stroke group showed greater phase error (*t*_|40|_= 3.52, *p* = 0.001; Cohen’s* d* = 1.09, Fig. 2D) and CV of phase (*t*_|40|_= 2.85, *p* = 0.007; Cohen’s* d* = 0.91, Fig. 2E) compared with the controls.

### Association between gait coordination and gait variability

Table 2 shows the relationship between gait variability and gait coordination measures in the stroke group. PCI was positively correlated to stride length variability (*r* = 0.49, *p* = 0.02) and stride time variability (*r* = 0.65, *p* < 0.001) (Table 2). The examination of correlations between stride length variability and stride time variability showed that the two variables were not highly correlated (*r* = 0.50). The collinearity statistics were within the accepted limits (*Tolerance* = 0.74, *Variance Inflation Factor* = 1.34), suggesting that the assumption of multicollinearity was met.

**Table 2:** Pearson’s bivariate correlations showing the relationship between phase coordination index (PCI), phase error, and coefficient of variation (CV) of phase and gait variability measures in the stroke group.

	Stride length CV			Stride time CV	
	*r*	*p*		*r*	*p*
PCI	**0.49**	0.024		**0.65**	0.001
Phase error	0.34	0.12		**0.50**	0.02
Phase CV	**0.53**	0.01		**0.65**	0.001

Significant *r* values are in bold.

**Figure 3:**
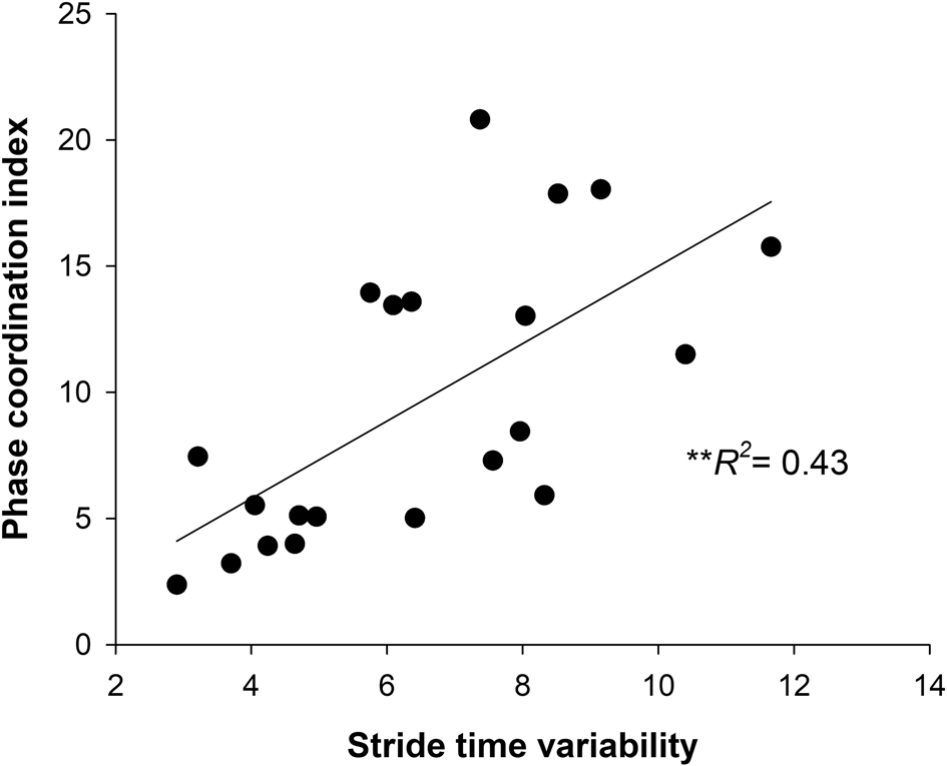
Predicting gait coordination between lower limbs from gait variability measures in stroke group: multiple regression analyses in the stroke group showed that only stride time variability was a significant predictor of phase coordination index. **p < 0.01.

The backward multiple regression performed to examine if the gait variability in the stroke group predicted PCI, showed that stride time variability (*β* = 0.65, *p* = 0.001), but not stride length variability (*β* = 0.21, *p* = 0.30), was a significant predictor of PCI (*R*^2^ = 0.43, *p* = 0.001; Fig. 3). Post-hoc power analysis showed that power of 91.60% was achieved with this sample.

### Association between accuracy and consistency of gait coordination and gait variability

**Figure 4:**
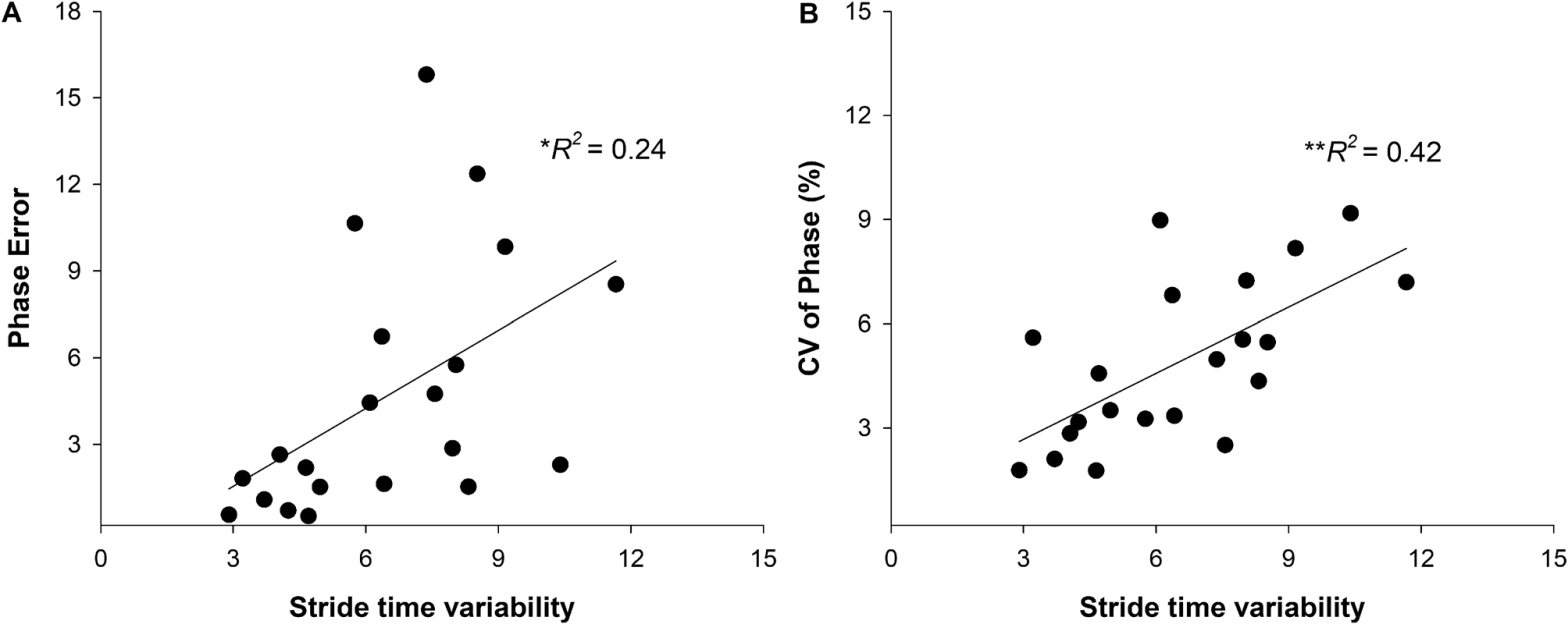
Predicting phase accuracy and consistency of phase from gait variability measures. Multiple regression analysis in the stroke group showed that stride length variability significantly predicted both (**A**) phase error and (**B**) CV of phase. Stride length variability did not emerge as a significant predictor of phase error and CV of phase in any of the regression analyses. *p < 0.05; **p < 0.01.

The stride length variability and stride time variability showed a significant positive relationship with phase error and CV of phase in the stroke group (Table 2). A backward multiple regression performed to determine if gait variability predicted the accuracy and consistency of gait coordination in stroke (*Variance Inflation Factor* = 1.34). The regression analyses revealed that only stride time variability (*β* = 0.49, *p* = 0.02) was a significant predictor of phase error (*R*^2^ = 0.24, *p* = 0.02; Fig. 4A). Similarly, only stride time variability (*β* = 0.65, *p* = 0.001) was a significant predictor of CV of phase (*R*^2^ = 0.42, *p* = 0.001; Fig. 4B). The stride length variability did not predict phase error (*β* = 0.12, *p* = 0.59) and CV of phase (*β* = 0.27, *p* = 0.18).

## Discussion

The current study investigated the effect of spatial and temporal gait variability on gait coordination between lower limbs during overground walking in chronic stroke survivors. The results suggest that increased stride time variability, but not stride length variability, contributes to gait incoordination in stroke survivors. Further, increased fluctuations in stride time directly impact the accuracy and consistency of gait coordination following stroke. We provide novel evidence that increased temporal variability in successive strides disrupts lower limb coordination during overground walking in stroke survivors. These findings have implications for development of targeted interventions that reduce gait variability to promote a stable walking pattern in stroke survivors.

Heightened motor variability is one of the most prominent impairments after stroke^32–37^. Recently, there has been considerable interest among scientists to understand the functional implications of increased motor variability on performance related outcomes in stroke survivors. In line with this motivation, here, we investigated the impact of gait variability on gait coordination to gain insights into how increased motor variability contributes to the lower limb incoordination during walking.

Rhythmic coordination between lower extremities is a fundamental element of stable walking and safe ambulation^19^. Commonly, gait deficits after stroke have been quantified using symmetry of limb kinematics rather than by examining the interaction between two legs. In the current study, we quantified lower limb coordination with PCI that indices the accuracy and consistency of generating the anti-phase leg movements during walking^13^. We found that ambulatory, chronic stroke survivors showed higher PCI indicating that lower limb coordination is significantly diminished during walking. These results are in line with previous studies showing inter-limb coordination deficits during voluntary motor tasks after stroke^15,21^. Furthermore, our stroke cohort showed 266.66% higher phase error and 48.78% lower phase consistency relative to the control group, suggesting that the impact of stroke is larger on accuracy rather than the consistency of the lower limb coordination required for walking. Such lower limb incoordination after stroke could impact the ability to adjust the walking pattern to environmental demands and lead to loss of balance.

Phase coordination index quantifies the inter-limb coordination in temporal domain by measuring step time relative to stride time. Post-stroke individuals adapt gait pattern to compensate sensorimotor impairments. For example, stroke survivors may decrease the stride length of the non-paretic to limit weight bearing on the weaker, paretic limb. Such intentional adaptation of stride length can affect the temporal parameters as the instance of heel contact is delayed or hastened with change in stride length^38^. From the biomechanical standpoint, inconsistency in both spatial and temporal gait parameters can alter gait coordination. Whether distinct neural mechanisms control spatial and temporal aspects of walking is not well understood. However, studies using split-belt and body-weight support treadmill training support that spatial and temporal strategies independently contribute to change in walking outcomes after stroke^39,40^. Further, spatial and temporal inter-limb coordination during overground walking has not been well investigated in stroke survivors. Only one study investigated inter-limb coordination during voluntary anti-phase ankle movements^21^. In this study stroke survivors had reduced ankle excursion and slow ankle movements resulting in impaired anti-phase coordination, highlighting that both spatial and temporal movement parameters affect lower limb coordination^21^. In the following sections, we discuss how increased gait variability in spatial and temporal domains contribute to deficits in gait coordination after stroke.

The most noteworthy finding of our study is that poor coordination between lower limbs during walking is associated with exacerbated stride time variability in stroke survivors. The PCI predominantly measures the temporal coordination of lower limbs. The stroke group showed greater stride length variability and stride time variability suggesting heightened inconsistency in timing and distance between consecutive strides. These findings are in line with previous reports showing increased gait variability in spatial and temporal domains following stroke^11,41^. We found that both stride length variability and stride time variability were positively correlated to PCI in the stroke group (Table 2). However, the regression analysis showed that only stride time variability was a significant predictor of PCI. Thus, the variability in timing of foot movement influences the lower limb incoordination to a greater degree than the variability in foot positioning during walking.

Increased stride time gait variability is consistently linked with mobility impairments, specifically increased fall risk in older adults and clinical populations^6,9,42–44^. Recent studies in stroke survivors found that compared with stride length variability, stride time variability contributed to a larger extent to the fear of falling and the amount of ambulatory activity^8,41^. Although stroke impacts both spatial and temporal stride variability, stride time variability may be more robust indicator of the decline in locomotor function. Furthermore, previous studies report that stroke survivors show better ability to modulate the stride length than stride time during walking^45,46^. Such reduction in the modulation of stride time may explain the association between temporal stride variability and coordination of lower extremities in stroke survivors. Further, we found that stride time variability independently predicted both phase error and CV of phase during overground walking in the stroke group. Thus, increased fluctuations in stride time directly impact the accuracy and consistency of gait coordination between lower limbs. These results further highlight that increased temporal gait variability is particularly detrimental through its impacts on both accuracy and consistency of gait coordination between lower limbs in stroke survivors. Prior work in older adults has confirmed that greater fall risk is associated with poor gait coordination between lower limbs^47^. Considering that increased temporal variability contributed to gait incoordination in our study, whether the link between gait variability and falls is mediated through poor gait coordination in stroke survivors constitutes a logical, next question for future investigation.

One of the limitations of our study is that the overground walking was completed over a relatively short distance of 14 m resulting in 10 to 32 strides per trial. A recent study indicated more than 23 strides are required to obtain a reliable estimation of PCI, however, this study was conducted in healthy young adults using overground and treadmill walking trials^48^. We intentionally selected multiple (three) shorter walking trials to ensure that all of our stroke participants were able to complete the walking trials without a break or without the use of a walking aid, that could indirectly influence gait variability. Moreover, averaging gait variability across three walking trials provided us a more conservative estimation of gait parameters and avoided artificial inflation due to a single spurious trial. Our rationale for shorter walking trials was supported by previous research indicating that as few as ten strides were sufficient to obtain gait variability measures with good reliability in individuals with pathological gait^26,49^. Regardless, future studies are needed to determine the number of strides required for reliable estimation of gait coordination in individuals with neurological conditions such as stroke. Gait variability between the paretic and non-paretic legs could differ in stroke survivors and may have distinct contribution to gait coordination, however, in our study gait variability of each leg was unavailable due to technical limitations of the equipment. Finally, our stroke cohort included relatively high functioning individuals (mean FMA-LE score 27.38 ± 6.08) who did not use a walking aid or orthoses for the walking task. Therefore, whether our results generalize to stroke survivors with sensorimotor impairments or individuals who use walking aids remains to be determined.

Each year, stroke survivors experience greater number of falls (25–35%) than their healthy counterparts (~ 11%), in spite of achieving community ambulation^50^. Previous studies suggest that despite recovery in gait speed and gait symmetry, ambulatory stroke survivors continue to experience mobility limitations and are more likely to fall than their healthy counterparts^12,50,51^. Our stroke cohort showed marked deficits in gait coordination (127.65% higher PCI relative to controls) despite achieving gait speeds associated with independent community ambulation (stroke group gait speed = 0.94 ± 0.29 m/s). Such differential recovery in gait speed and gait coordination underscores the need to incorporate gait coordination as a critical rehabilitation target for restoring safe ambulation after stroke. Recent studies provide some evidence that force-control and dual-task walking trainings may be effective in reducing gait variability after stroke^30,52^. Accordingly, our findings have direct implications on the development of interventions targeting gait variability to improve coordination during walking after stroke.

In conclusion, impaired lower limb coordination during walking in ambulatory, chronic stroke survivors is associated with exacerbated stride time variability, but not stride length variability. Increased stride time variability impairs gait coordination by impacting both accuracy and consistency of anti-phase coordination of lower limbs. These findings highlight that heightened temporal gait variability negatively impacts the quality of gait coordination in stroke survivors. Targeted motor interventions that reduce temporal gait variability could improve lower limb coordination during walking and promote stable walking in individuals with stroke.

## Methods

### Participants

Twenty-one ambulatory, chronic stroke survivors and 21 healthy controls participated in this study. The characteristics of each participant are shown in Table 1. The inclusion criteria for the individuals with stroke were: (1) diagnosed with a stroke at least 6 months before study enrollment, (2) ability to ambulate independently with or without a walking aid, and (3) ability to follow a three-step command. Participants were excluded if they presented (1) any neurological disorder other than stroke, (2) musculoskeletal impairments, pain or injury in the lower limbs (3) uncorrected visual or hearing impairments, and (4) global aphasia. All participants read and signed an informed consent approved by the Institutional Review Board of University of Florida before participating in the study. All procedures were performed in accordance with guidelines from Institutional Review Board of University of Florida.

### Clinical assessments

In the stroke group, we performed the Fugl-Meyer Assessment (FMA) to determine the degree of motor impairments in the lower extremities. Across all participants, Frenchay Activities Index (FAI) measured the level of participation in instrumental activities of daily living.

### Overground walking

**Figure 5:**
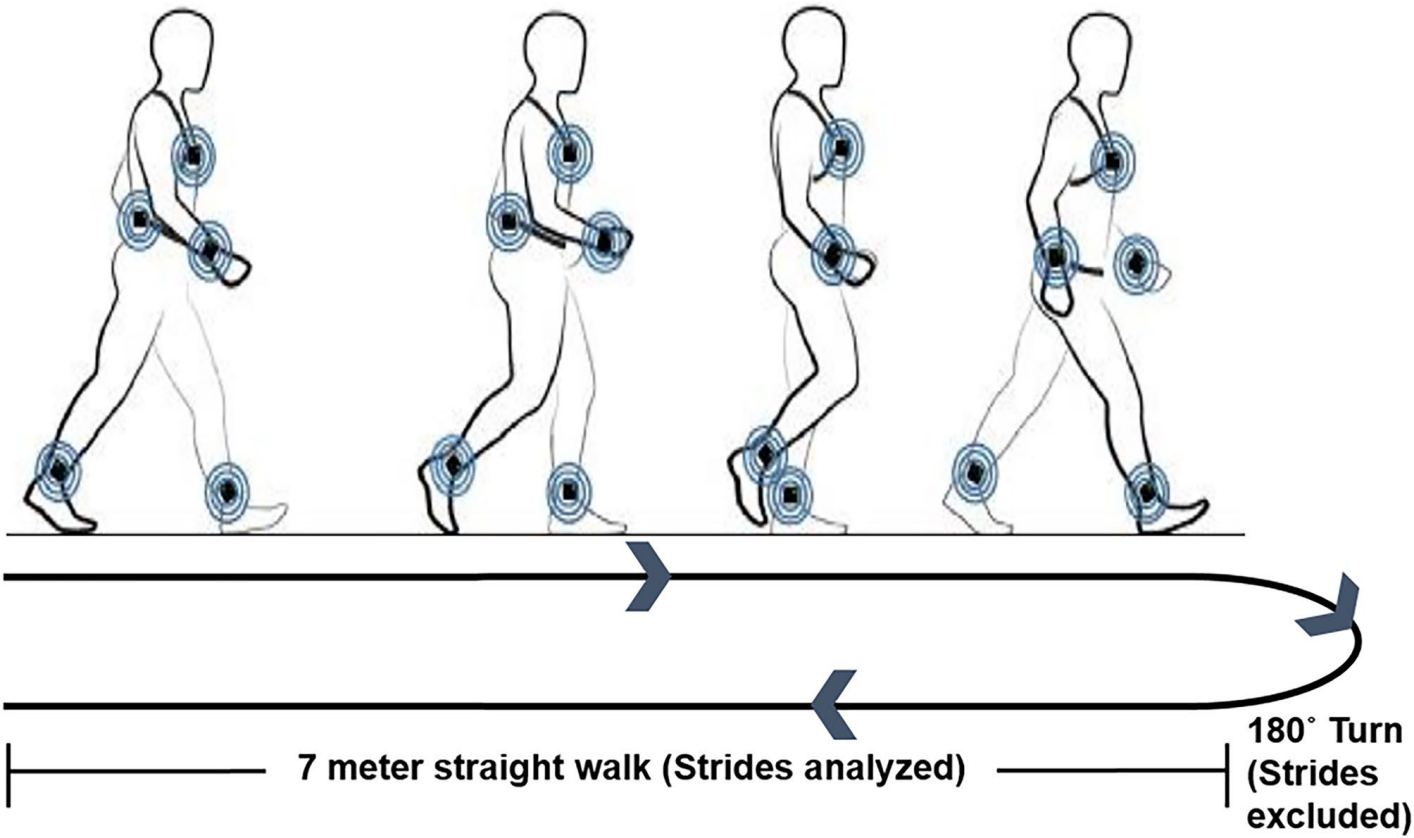
Schematic of the walking task: Participants walked straight for 7 m then turned 180° and walked back straight 7 m. Consecutive strides from the straight portion of the walking trial were used to compute the gait variables and the strides involved in turning were excluded from the gait analysis.

As shown in Fig. 5, the walking task involved walking for seven meters straight, turning around after the seven meter mark, and walking back seven meters straight to the starting point. Participants were instructed to walk at their preferred walking speed. Three trials were performed with a 90-s rest period between each trial. All participants walked without the use of walking aids or orthoses. The gait data were measured using six wireless inertial sensors (Opal, APDM Wearable Sensor Technologies. Inc, Portland, OR, USA) worn by the participant. The six inertial sensors were located on the two wrists, two ankles, sternum, and lumbar region. The position and acceleration data from the sensors were used to identify gait events and measure gait kinematics using the Mobility Lab software (APDM Wearable Sensor Technologies. Inc, Portland, OR, USA). The gait data were validated after each trial and stored for offline analyses. The wireless inertial sensors have been identified as a valid and reliable method for recording gait kinematics in individuals with gait disorders^53,54^.

### Data analysis

#### Gait variability

The stride length variability was quantified as the coefficient of variation (CV) of stride length for each walking trial. Similarly, the stride time variability was measured as the CV of stride time. The CV of stride length and stride time were calculated over the cumulative means for each consecutive gait cycle.

#### Phase coordination index (PCI)

The temporal gait parameters including step time (i.e. time between heel strike of one leg to the heel strike of the other leg) and stride time (i.e. from heel strike on one leg to the next heel strike of the same leg) were measured for each gait cycle using the gait events. In a single walking trial, the phase (Φ) value for each gait cycle was calculated by dividing the step time by stride time and multiplying by 360˚ (Eq. 1). To maintain the consistency in measuring the phase for a specific leg across all participants, we first determined the average swing time for each leg within a single walking trial. Then, we used the leg with longer average swing time as the reference leg for gait cycle and measured the phase values for the opposite leg^13^. The absolute mean phase value was calculated across all the gait cycles. The phase error was measured as the absolute mean phase error relative to the ideal phase of 180˚ (Eq. 2). The consistency of phase across all the gait cycles was measured as the coefficient of variation (CV) of phase values (Eq. 3). The phase coordination index (PCI) for each gait trial was calculated by summating both the phase error and the CV of phase. A higher PCI value is indicative of poorer coordination.

1 $$\Phi i= \frac{{t}_{si}-{t}_{Li}}{{t}_{L\left(i+1\right)}-{t}_{Li}} \times 360^\circ$$

where t_*Li*_ and t_*Si*_ denote the time of the *i*th heel strike of the legs with longer mean swing time and mean shorter swing times respectively, and t_*L(i* + *1)*_ > t_*Li*_ > t_*Si*_.

2 $$\Phi Error=\frac{Abs(Mean \,Phase-180)}{180} \times 100^\circ$$

3 $$CV of \Phi =\left(\frac{Standard \, Deviation \, of \, Phase}{Mean \,Phase}\right)\times 100$$

The gait speed was computed using the time taken to walk 14 m. The number of gait cycles for each trial ranged from 10 to 32 based on participant’s walking speed. We removed the gait cycles related to turning for PCI and gait variability measurement (Fig. 5). All gait variables were measured for each trial and averaged across three trials.

### Statistical analysis

The Shapiro Wilk test confirmed the normality of the data for gait variability and coordination outcome measures. We compared the spatial and temporal gait variability, PCI, phase error, and CV of phase between the groups with independent *t*-tests and calculated the effect size with Cohen’s *d*. Pearson’s bivariate correlations examined the relationship between stride length variability, stride time variability, PCI, phase error, and CV of phase for the stroke group. To determine the contribution of temporal and spatial gait variability to gait coordination in the stroke group, we performed backward stepwise multiple regression with stride length variability and stride time variability as predictor variables and PCI as the criterion variable. Given that PCI is a composite measure of phase accuracy and consistency, we performed two separate regression analyses to predict phase error and CV of phase from gait variability measures to further understand whether gait variability differentially influences one (accuracy or consistency) or both aspects of gait coordination. All the analyses were performed with SPSS 24.0 (IBM Corp) with α set at 0.05.

### Ethics approval and consent to participate

All participants read and signed an informed consent form prior to participation in the study. The study procedures and the consent form were approved by the institutional review board of University of Florida.

## Data Availability

Data used to support study findings are included in the manuscript. Additional data can be provided up on a reasonable request to the corresponding author.
